# Anti-Aging Effects of Some Selected Iranian Folk Medicinal Herbs-Biochemical Evidences

**Published:** 2013-11

**Authors:** Azadeh Mohammadirad, Fatemeh Aghamohammadali-Sarraf, Simin Badiei, Zakie Faraji, Reza Hajiaghaee, Maryam Baeeri, Mahdi Gholami, Mohammad Abdollahi

**Affiliations:** 1 Department of Toxicology and Pharmacology, Faculty of Pharmacy, and Pharmaceutical Sciences Research Center, Tehran University of Medical Sciences (TUMS), Tehran, Iran; 2 Pharmaceutical Sciences Branch, Islamic Azad University, Tehran, Iran; 3 Pharmacognosy & Pharmaceutics Department of Medicinal Plants Research Center, Institute of Medicinal Plants, ACECR, Karaj, Iran

**Keywords:** Aging, D-galactose, Herbal, Mouse, Oxidative stress

## Abstract

***Objective(s):*** In the current study, the effects of selected folk medicinal herbs were evaluated in D-galactose-induced aging in male mice.

***Materials and Methods:*** Male BALB/c mice were randomly divided into 12 groups composing sham, control, and treated groups. Aging was induced by administration of D-galactose (500 mg/kg/day for 6 weeks). A positive control group was assigned that received vitamin E (200 mg/kg/day). The extract of herbs was prepared, lyophilized, and used in this study. The herbs were administered by gavage for 4 weeks to D-galactose-aged animals at the selected doses (mg/kg/day) as follows: *Zingiber officinale* (250), *Glycyrrhiza glabra* (150), *Rosmarinus officinalis* (300), *Peganum harmala* (50), *Aloe vera* (150), *Satureja hortensis* (200), *Teucrium scordium *(200), *Hypericum perforatum* (135) and *Silybum marianum* (150). One group of animals was assigned as sham and not given D-galactose.

***Results:*** At the end of treatment, pro-inflammatory markers including tumor necrosis factor-α (TNF-α), interlukine-1β (IL-β), interlukine-6 (IL-6), NF-kappaB (NF-κb), total antioxidant power (TAP), thiobarbituric acid reactive substances (TBARS) as lipid peroxidation (LPO) marker and male sex hormones i.e. testosterone and dehydroepiandrosterone-sulfate (DHEA-S) were measured in the blood.

***Conclusion:*** These data for the first time indicate significant anti-aging potential of examined herbs. Results showed that D-galactose induces a significant oxidative stress and promotes proinflammatory cascade of aging while all herbs more or less recovered these changes. Among 9 herbal extracts, Silybum marianum showed the best effect in restoring aging changes.

## Introduction

Aging as a complex of natural circumstance is exhibited by an augmentation in the chance of illness and finally death. Although some theories have been proposed as the mechanisms of aging but the one relating aging and cellular oxidative stress have received more supports. Therefore, it can be said that reduced sex hormones and augmented quantity of oxidative stress parameters or inflammatory cytokines are main biochemical manifestations of aging (1-2). In accord with this theory, the production of reactive oxygen species (ROS) and/or free radicals can injure cells and tissues paralleled by malfunction of many systems. The eventual consequence of these actions is aging and finally premature cell death (3). During aging process, various pro-inflammatory molecules are generated to strengthen inflammation cascade associated with different age-related pathologies (4). 

One of the problems in testing anti-aging compounds is lack of suitable animal models. Although several models have been used so far but among them, typical mouse D-galactose-induced model of aging is the best one that gives closer results to clinical studies. D-galactose is a sugar that at higher levels converts to aldose and hydro-peroxide during the catalysis of galactose oxidase, culminated in the generation of free radicals (6). These modifications are substantially similar to the normal aging process demonstrated as neurological deterioration, diminished activity of antioxidant enzymes, and miserable immune responses (7-8).

Many scientists and pharmaceutical companies try to develop a drug to reduce speed of human aging but no effective drug has been discovered yet. In the last decade the importance of folk medicine and herbal medicines have been revisited that resulted in developing many effective drugs for many human diseases. For instance, in the recent years, efficacy of herbal medicines in diseases like inflammatory bowel diseases (9-10), obesity (11), diabetes (12), pancreatitis (13), osteoporosis (14), hyperlipidemia (15), and so on has been proved. Our recent systematic review specified anti-aging herbs and their characteristics in different clinical or experi-mental models (16). Most of anti-aging herbs have antioxidant components and reduces free radicals which are by-product of abnormal body metabolism in the elderly. 

We recently proved anti-aging potential of naturally-based drugs like IMOD and Angipars which have strong antioxidant power (2). On the basis of our systematic review, among various species we could select nine herbs with the strongest antioxidant effects such as *Z. officinale, G. glabra*, *R. officinalis, P. harmala, A. vera,* *S. hortensis*, *T. scor-dium*, *H. perforatum* and *S. marianum* to test in D-galactose-induced model of mouse aging.

## Materials and Methods

*Chemicals*

Thiobarbituric acid (TBA), trichloroacetic acid (TCA), n-butanol, hexadecyltrimethyl ammonium bromide (HETAB), tri (2-pyridyl)-s-triazine (TPTZ), HCl, malondialdehyde (MDA), ferric chloride (FeCl_3_-6H_2_O), D-galactose, and vitamin E (Trolox) were purchased from Merck (Germany). Rat specific tumor necrosis factor-α (TNF-α), interlukine-1β (IL-β), interlukine-6 (IL-6), NF-kappa B (NF-κb) ELISA kits were purchased from BenderMed Systems (Austria). Testosterone and dehydroepiandrosterone ELISA kits were purchased from Dia Metra (Italy). 

*Preparation of herbs, extraction, and lyophilization*

Herbs were provided from the Research Institute of Medicinal Plants Karaj during June 2009 and were air-dried at room temperature. Samples were authenticated by a botanist (Y. Ajani), and voucher specimens were preserved in the central herbarium of medicinal plants (RIMP). The scientific names and tested parts of the herbal materials are detailed in Table 1. The dried plants powder (40 g) was extracted using percolation method by methanol at room temperature. Solvents were completely removed by drying under reduced pressure at 40°C in a rotary evaporator. The samples were stored at 4°C until use. Specifically, the *A. vera* leaves (1000 g) were washed in a suitable bactericide (chlorh-exidine). The filets were grounded to a liquid, and the pulp was removed by filtering. The resultant gel was then freeze dried.

*Animals*

Male BALB/c mice (12 weeks old, 18–22 g) were provided from Tehran University of Medical Sciences (TUMS) animal house. The animals were housed in standard polypropylene cages with wired-net top in a controlled room (temperature 23±1°C, humidity 55±10%, 12 hr light–dark cycle) and were allowed free access to standard laboratory pellet diet and water during the experiments. All ethical issues on the use of animals were carefully considered and the study protocol was approved by TUMS review board with code number of 90-03-33-15668.

*Experimental design*

Before starting the main study, a pilot was designed to set up aging model and to get proper doses of herbal materials. In the main study, 120 mice were randomly divided into 12 groups, each consisting of 10 animals. D-galactose was dissolved in a measured quantity of mice drinking water. D-galactose was given to 11 out of 12 groups of animals at 500 mg/kg D-galactose per 1 ml drinking water for 6 weeks by gavage (2, 17). The 12^th^ group of animals was the sham group which was not given D-galactose. After 2 weeks, the 11 groups which had been given D-galactose were randomly divided into aging control group (500 mg/kg D-galactose per 1 ml drinking water, for 6 weeks), positive control group (500 mg/kg D-galactose per 1ml drinking water plus vitamin E 200 mg/kg/day by gavage for 4 weeks) and herb-treated groups including 9 groups that each received 500 mg/kg D-galactose per 1 ml drinking water plus *Z. officinale* (250 mg/kg/day), *G. glabra* (150 mg/kg/day), *R. officinalis* (300 mg/kg/day), *P. harmala* (50 mg/kg/day), *A. vera* (150 mg/kg/day), *S. hortensis* (200 mg/kg/day), *T. scordium* (200 mg/kg/day), *H. perforatum* (135 mg/kg/day) and *S. marianum* (150 mg/kg/day), respectively by gavage for 4 weeks (18-26).

**Table 1:** The scientific names and tested parts of the plant materials

Scientific name	Tested parts	Extraction yield (mg/g)	Used Dose (mg/kg)	References
*Zingiber officinale*	Rhizome	140.57	250	18
*Glycyrrhiza glabra*	Root	129.52	150	19
*Rosmarinus officinalis*	Aerial parts	236.51	300	20
*Peganum harmala*	Seed	169.25	50	21
*Aloe vera*	Gel	4.87	150	22
Satureja hortensis	Aerial parts	134	200	23
Teucrium scordium	Aerial parts	205	200	24
*Hypericum perforatum*	Aerial parts	100.58	135	25
Silybum marianum	Seed	123.49	150	26

Twenty-four hours after the last treatment, blood samples were taken of each animal under anesthesia through the tail vein. Serum samples were obtained by centrifuging the whole blood at 1000 × g at 4°C for 10 min and the supernatants were transferred into several microtubes for separate biochemical assays and maintained at -80°C until the analyses were performed. Biochemical markers including TNF-α, IL-β, IL-6, NF-κb, ferric reducing total antioxidant power (TAP), lipid peroxidation (LPO) and male sex hormones including testosterone and dehydroepiandrosterone-sulfate (DHEA-S) were measured in the serum.

*Measurement of LPO*

LPO was measured by the reaction of thiobarbituric acid (TBA) with lipid peroxides. Samples were mixed with TCA (20%) and the precipitate was dispersed in H_2_SO_4_ (0.05 M). After addition of TBA (0.2% in sodium sulfate), the sample was heated for 30 min in a boiling water bath. Then, TBA reactive substances (TBARS) as LPO marker adducts were extracted by n-butanol and absorbance was measured at 532 nm as described in details in our previous work (27). Data were expressed as nM.

*Measurement of TNF-α, IL-1β, IL-6 and NF-κb*

Quantitative detection of TNF-α, IL-1β, IL-6 and NF-κb levels in serum were performed using an enzyme-linked immunosorbent assay rat specific ELISA kit according to each specific brochure. The absorbance of the final colored product was measured in 450 nm as the primary wave length and 620 nm as the reference wave length. TNF-α, IL-1β, IL-6 and NF-κb levels were expressed as pg/mg.

*Measurement of TAP*

Serum TAP was evaluated by measuring the ability to reduce Fe^3+^ to Fe^2+^. Interaction of TPTZ with Fe^2+^ results in formation of a blue color with a maximum absorbance at 593. The whole procedure has been described in our previous study (27). Data were expressed as mM.

*Measurement of testosterone and DHEA-S*

For determination of testosterone and DHEA-S, specific ELISA kits were used and the instruction of their brochure was followed. Testosterone and DHEA-S were expressed as ng/ml.

*Statistical analysis*

Results are expressed as mean±standard error of the mean (SEM). Data were analyzed by one-way ANOVA followed by Tukey post-hoc test for multiple comparisons to ensure the variances of the data are distributed properly. A *P*-value less than 0.05 were considered significant. The Stats Direct version 2.7.9 was used. 

## Results

**Figure 1:**
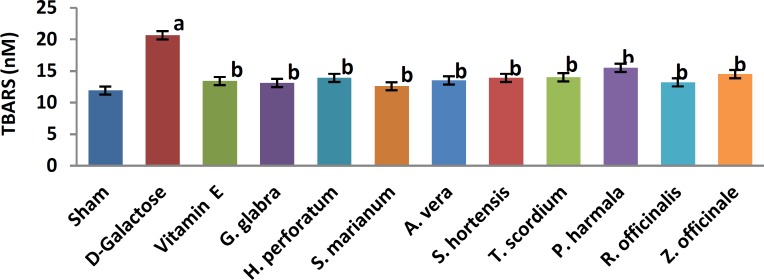
Effects of herbs on serum thiobarbituric acid reactive substances (TBARS) as lipid peroxidation (LPO) marker in aged mice. Data are mean±SEM of ten animals. ^a^Significantly different between sham group and other groups at *P*<0.05. ^b^Significantly different between D-galactose group and other groups at *P*<0.05

**Figure 2:**
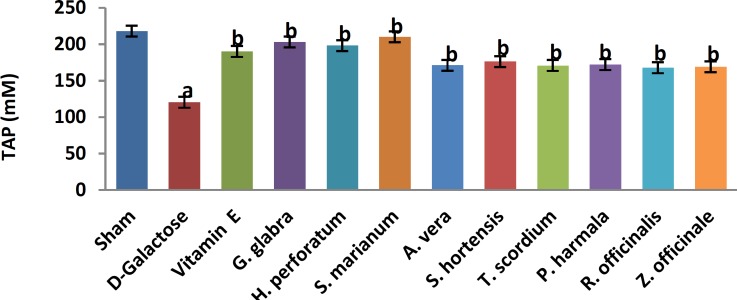
Effects of herbs on serum total antioxidant power (TAP) of aged mice. Data are mean±SEM of ten animals ^a^Significantly different between sham group and other groups at *P*<0.05 ^ b^Significantly different between D-galactose group and other groups at *P*<0.05

**Figure 3:**
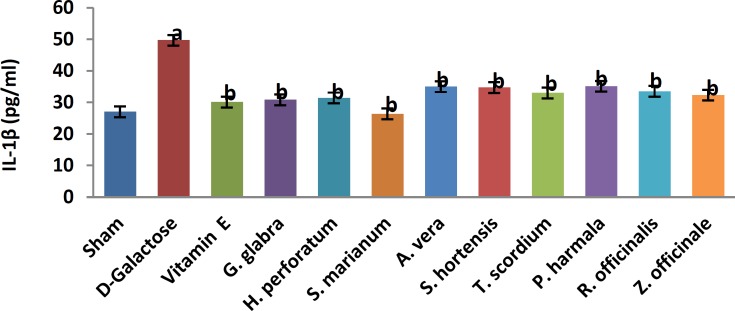
Effects of herbs on serum interleukin-1 beta (IL-1β) in aged mice. Data are mean±SEM of ten animals ^a^Significantly different between Sham group and other groups at *P*<0.05 ^b^Significantly different between D-Galactose group and other groups at *P*<0.05

**Figure 4:**
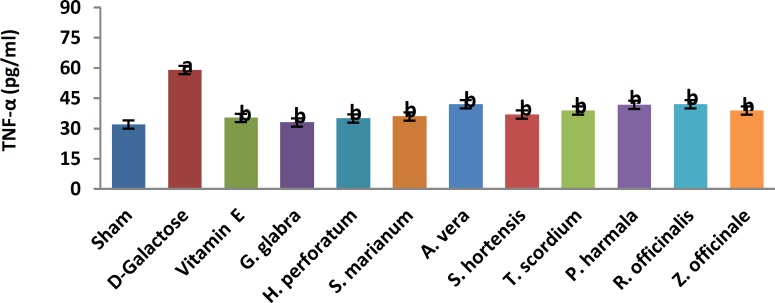
Effects of herbs on serum tumor necrosis factor-alpha (TNF-α) in aged mice. Data are mean±SEM of ten animals ^a^Significantly different between Sham group and other groups at *P*<0.05 ^b^Significantly different between D-Galactose group and other groups at *P*<0.05.

**Figure 5:**
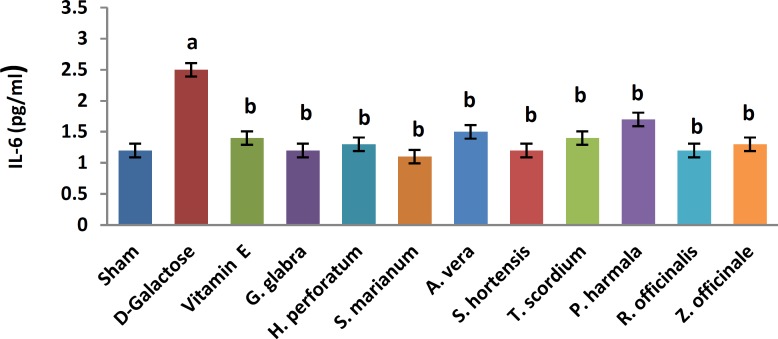
Effects of herbs on serum interleukin 6 (Il-6) in aged mice. Data are mean±SEM of ten animals ^a^ Significantly different between Sham group and other groups at *P*<0.05 ^b^Significantly different between D-Galactose group and other groups at *P*<0.05

**Figure 6:**
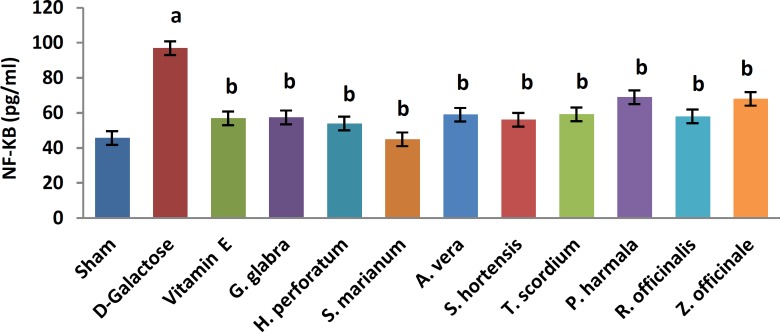
Effects of herbs on serum NF-kappaB (NF-kB) in aged mice. Data are mean±SEM of ten animals ^a^Significantly different between Sham group and other groups at *P*<0.05 ^b^Significantly different between D-Galactose group and other groups at* P*<0.05

**Figure 7:**
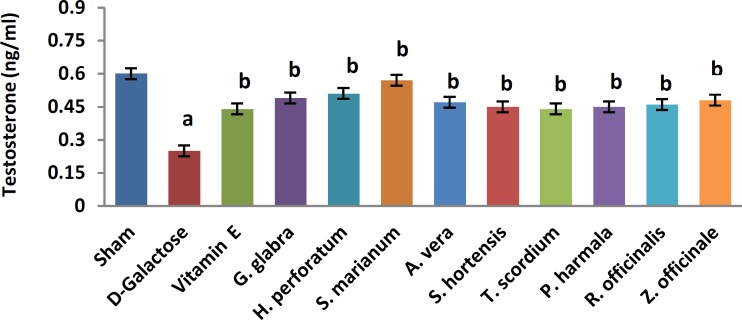
Effects of herbs on serum testosterone in aged mice. Data are mean±SEM of ten animals ^a^Significantly different between Sham group and other groups at *P*<0.05 ^b^Significantly different between D-Galactose group and other groups at *P*<0.05

**Figure 8:**
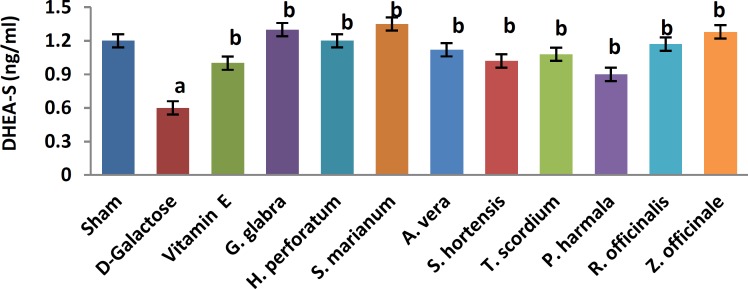
Effects of herb on serum dehydroepiandrosterone-sulfate (DHEA-S) in aged mice. Data are mean±SEM of ten animals ^a^Significantly different between Sham group and other groups at *P*<0.05 ^b^Significantly different between D-Galactose group and other groups at *P*<0.05

A significant increase in TBARS (Figure 1, 11.9±0.2 vs. 20.66±0.88, *P*< 0.05) and a significant decrease in TAP (Figure 2, 218±8 vs. 120±7.5, *P*<0.05) were observed when sham group was compared with D-galactose-received aged group. Figures 3-6 show the effects of aging on the levels of TNF-α, IL-6, IL-1β, and NF-kB, respectively in comparison to sham (32±2.3 vs. 59±15, *P*<0.05; 1.2±0.05 vs. 2.5±0.33, *P*<0.05; 27±3.9 vs. 49.66±3.4, *P*<0.05; 45.7±2.4 vs. 97±21.2, *P*<0.05). As shown in Figures 7 and 8, testosterone and DHEA-S (0.6±0.05 vs. 0.25±0.03, *P*<0.05; 1.2±0.2 vs. 0.6±0.08, *P*<0.05) in aged mice was lower than that in the sham.

*Effects of* *Z. officinale* *in aged mice*

*Z. officinale* treatment recovered D-galactose-induced rats by reducing TBARS (14.5±1.6 vs. 20.66±0.88, *P*<0.05), and increasing TAP (169±3.5 vs. 120±7.5, *P*<0.05), (Figures 1, 2). Figures 3-6 show that administration of *Z. officinale* recovered D-galactose-induced increase in TNF-α, IL-6, IL-1β, and NF-kB (39±2.6 vs. 59±15, *P*<0.05; 1.3±0.3 vs. 2.5±0.33, *P*<0.05; 32.3±0.54 vs. 49.66±3.4, *P*<0.05; 68.1±5.7 vs. 97±21.2, *P*<0.05), respectively. As shown in Figures 7 and 8, *Z. officinale* increased testosterone and DHEA-S (0.48±0.04 vs. 0.25±0.03, *P*<0.05; 1.28±0.17 vs. 0.6±0.08, *P*<0.05) in aged mice.

*Effects of* *G. glabra* *in aged mice*

D-galactose-induced elevation of TBARS and reduction of TAP (Figures 1, 2) were significantly recovered following treatment with *G. glabra *(13.1±1.01 vs. 20.66±0.88, *P*<0.05; 203±17 vs. 120±7.5, *P*<0.05).Figures 3-6show that adminis-tration of *G. glabra* recovered D-galactose-induced increase in TNF-α, IL-6, IL-1β and NF-kB (33±9.5 vs. 59±15, *P*<0.05;1.2±0.14 vs. 2.5±0.33, *P*<0.05; 30.78±3.1 vs. 49.66±3.4, *P*<0.05; 57.52±8.7 vs. 97±21.2, *P*<0.05), respectively. As shown in Figures 7 and 8, *G. glabra* increased testosterone and DHEA-S levels (0.49±0.05 vs. 0.25±0.03, *P*<0.05; 1.3±0.34 vs. 0.6±0.08, *P*<0.05) in aged mice.

*Effects of* *R. officinalis* *in aged mice*

*R. officinalis *treatment recovered D-galactose-induced rats by reducing TBARS (13.2±0.63 vs. 20.66±0.88, *P*<0.05), and increasing TAP (167.7±5.3 vs. 120±7.5, *P*<0.05) (Figures 1, 2). Figures 3-6 show that administration of *R. officinalis* recovered D-galactose-induced increase in TNF-α, IL-6, IL-1β, and NF-kB (42±12 vs. 59±15, *P*<0.05; 1.2±0.2 vs. 2.5±0.33, *P*<0.05; 33.5±4.1 vs. 49.66±3.4, *P*<0.05; 58.1±3.8vs. 97±21.2, *P*<0.05), respectively. As shown in Figures 7 and 8, *R. officinalis* increased testosterone, (0.46±0.09 vs. 0.25±0.03, *P*<0.05) and DHEA-S (1.17±0.19 vs. 0.6±0.08, *P*<0.05) in aged mice.

*Effects of* *P. harmala* *in aged mice*

D-galactose-induced elevation of TBARS and reduction of TAP (Figure 1, 2) were significantly recovered following treatment with *P. harmala* (15.5±1.73 vs. 20.66±0.88, *P*<0.05; 172±13.9 vs. 120±7.5, *P*<0.05). Figures 3-6 show that admin-istration of *P. harmala* recovered D-galactose-induced increase in TNF-α, IL-6, IL-1β, and NF-kB (41.8±6.8 vs. 59±15, *P*<0.05; 1.7±0.18 vs. 2.5±0.33, *P*<0.05; 35.1±1.42 vs. 49.66±3.4, *P*<0.05; 69±7.2 vs. 97±21.2, *P*<0.05), respectively. As shown in Figures 7 and 8, *P. harmala* increased testosterone and DHEA-S (0.45±0.08 vs. 0.25±0.03, *P*<0.05; 0.9±0.07 vs. 0.6±0.08, *P*<0.05) in aged mice.

*Effects of* *A. vera* *in aged mice*

*A. vera* treatment recovered D-galactose-induced elevation of TBARS (Figure 1, 13.5±1.7 vs. 20.66±0.88, *P*<0.05), and improved reduction of TAP (Figure 2, 171±4.03 vs. 120 ± 7.5, *P*< 0.05). Figures 3-6 show that administration of *A. vera* recovered D-galactose-induced increase in TNF-α, IL-6, IL-1β, and NF-kB (42±6.94 vs. 59±15, *P*<0.05; 1.5±0.09 vs. 2.5±0.33, *P*<0.05; 35±0.77 vs. 49.66±3.4, *P*<0.05; 59±14 vs. 97±21.2, *P*<0.05), respectively. As shown in Figures 7 and 8, *A. vera *increased testosterone and DHEA-S levels (0.47±0.09 vs. 0.25±0.03, *P*<0.05; 1.12±0.19 vs. 0.6±0.08, *P*<0.05) in aged mice.

*Effects of* *S. hortensis* *in aged mice*

*S. hortensis* treatment recovered D-galactose-induced elevation of TBARS (Figure 1, 13.9±2.2 vs. 20.66±0.88, *P*<0.05), and increased TAP (Figure 2, 176±14.16 vs. 120±7.5, *P*<0.05). Figures 3-6 show that administration of *S. hortensis* recovered D-galactose-induced increase in TNF-α, IL-6, IL-1β, and NF-kB (37±9.9 vs. 59±15, *P*<0.05; 1.2±0.05 vs. 2.5±0.33, *P*<0.05; 34.7±1.8 vs. 49.66±3.4, *P*<0.05; 56.2±13.9 vs. 97±21.2, *P*<0.05), respectively. As shown in Figures 7 and 8, *S. hortensis* increased testosterone and DHEA-S (0.45±0.05 vs. 0.25±0.03, *P*<0.05; 1.02±0.15 vs. 0.6±0.08, *P*<0.05) in aged mice.

*Effects of* *T. scordium* *in aged mice*

D-galactose-induced elevation of TBARS and reduction of TAP (Figure 1, 2) were significantly recovered following treatment with *T. scordium* (14±0.76 vs. 20.66±0.88, *P*<0.05; 170.8±7.64 vs. 120±7.5, *P*<0.05). Figures 3-6 show that adminis-tration of *T. scordium* recovered D-galactose-induced increase in TNF-α, IL-6, IL-1β, and NF-kB (39±10.26 vs. 59±15, *P*<0.05; 1.4±0.28 vs. 2.5±0.33, *P*<0.05; 33±1.1 vs. 49.66±3.4, *P*<0.05; 59.3±4.42 vs. 97±21.2, *P*<0.05), respectively. As shown in Figures 7 and 8, *T. scordium* increased testosterone and DHEA-S (0.44±0.05 vs. 0.25±0.03, *P*<0.05; 1.08±0.24 vs. 0.6±0.08, *P*<0.05) in aged mice.

*Effects of* *H. perforatum* *in aged mice*

*H. perforatum* treatment recovered D-galactose-induced rats by reducing TBARS (13.9±1.9 vs. 20.66±0.88, *P*<0.05) and increasing TAP (198±23 vs. 120±7.5, *P*<0.05) (Figures 1, 2). Figures 3-6 show that administration of *H. perforatum *recovered D-galactose-induced increase in TNF-α, IL-6, IL-1β and NF-kB (35±6 vs. 59±15, *P*<0.05; 1.3±0.17 vs. 2.5±0.33, *P*<0.05; 31.4±0.51 vs. 49.66±3.4, *P*<0.05; 53.98±2.7 vs. 97±21.2, *P*<0.05), respectively. As shown in Figures 7 and 8, *H. perforatum* increased testosterone and DHEA-S (0.51±0.06 vs. 0.25±0.03, *P*<0.05; 1.2±0.18 vs. 0.6±0.08, *P*<0.05) in aged mice.

*Effects of* *S. marianum* *in aged mice*

D-galactose-induced elevation of TBARS and reduction of TAP (Figure 1, 2) were significantly recovered following treatment with *S. marianum* (12.58±0.64 vs. 20.66±0.88, *P*<0.05; 210±12.14 vs. 120±7.5, *P*<0.05). Figures 3-6 show that adminis-tration of S. marianum recovered D-galactose-induced increase in TNF-α, IL-6, IL-1β, and NF-kB (36±5.4 59±15, *P*<0.05; 1.1±0.09 vs. 2.5±0.33, *P*<0.05; 26.36±1.1 vs. 49.66±3.4, *P*<0.05; 45±4.2 vs. 97± 21.2, *P*<0.05), respectively. As shown in Figures 7 and 8, the *S. marianum* recovered D-galactose-induced reduction of testosterone and DHEA-S (0.57±0.09 vs. 0.25±0.03, *P*<0.05; 1.35±0.22 vs. 0.6±0.08, *P*<0.05) in aged mice.

*Effects of* *vitamin E* *in aged mice*

D-galactose-induced elevation of TBARS and reduction in TAP (Figure 1, 2) were significantly recovered following treatment with Vitamin E (13.4±0.83 vs. 20.66±0.88, *P*<0.05; 190±13.1 vs. 120±7.5, *P*<0.05). Figures 3-6 show that administration of Vitamin E recovered D-galactose-induced increase in TNF-α, IL-6, IL-1β, and NF-kB (35.5±2.12 vs. 59±15, *P*<0.05; 1.4±0.26 vs. 2.5±0.33, *P*<0.05; 30.1±2.2 vs. 49.66±3.4, *P*<0.05; 57±3.9 vs. 97±21.2, *P*<0.05), respectively. As shown in Figures 7 and 8, vitamin E increased testosterone and DHEA-S (0.44±0.02 vs. 0.02±0.03, *P*<0.05; 1±0.16 vs. 0.6±0.08, *P*<0.05) in aged mice.

## Discussion

In this study, for the first time, we analyzed the anti-aging potentials of nine famous herbs in a well-setup animal aging model using chronic administration of D-galactose. Our results showed that production of free radicals is the principal reason of up-regulation of pro-inflammatory cytokines and the main determinant involved in the D-galactose-induced aging model. Furthermore, these herbs dramatically diminished oxidative stress and proinflammatory cytokines in the aged mice. Supporting the mechanism of action of these herbs and the theory of oxidative stress in aging, vitamin E was used as the standard and showed the similar effects in examined markers of aging.

Adapted from corresponding author’s previous paper published in open access source (16).

**Figure 9:**
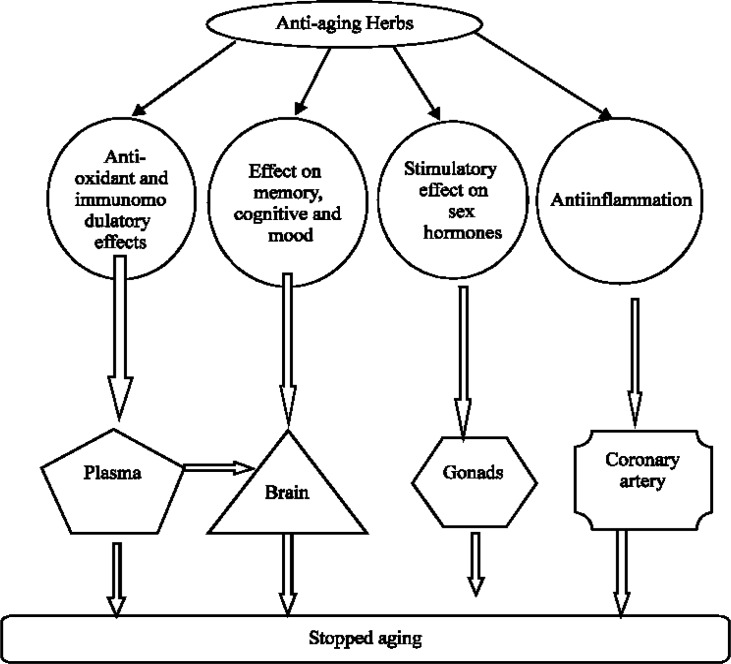
Suggested mechanisms of action of herbs in reducing aging process

Interestingly, present results indicated improve-ment of testosterone and DHEA-S by herbs in the aged mice. Decline of steroid hormones with aging is already known and is believed a major contributor to elevation of pro-inflammatory markers (28).

Recent studies have shown the mechanisms of action of anti-aging herbs in reducing aging process that is divided into four categories including anti-oxidant, anti-inflammatory, effect on memory/cog-nition/mood, and the sex hormones (Figure 9). This indicates that most of anti-aging herbals have antioxidant components (16) and thus supports the present findings and hypothesis of this study. 

*Ginger [Zingiber officinale Roscoe (*Zingiberaceae*)]* and supplements derived from ginger like zingerone, shogaols and gingerols posse the abilities for the treatment of chronic inflammation. The protective effects of *Z. officinale* in lessening macromolecular damage in aged mice were shown in this study. Besides, recent study has shown that ginger extracts owns antioxidant activity (29). It has been recently shown that pre-trial administration of this herb expedites conditioned inhibitory learning in adult rats (30). Also, it has been found that *Z. officinale *has possibly good effects on age-related execution shortages and defends against oxidative stress in old rats, suggesting this compound as a useful factor in treating age-related disturbances (31).

*G. glabra* (licorice extract) or licorice is the root of *G. glabra* from which a sweet flavor can be extracted. The results of this study showed that *G. glabra* has the protective effects in declining macromolecular damage in aged mice. It has been shown that *G. glabra* extract is the safest pigment-lightening agent with the fewest side effects (32). Additionally, *G. glabra* has anti-inflammatory properties hypothe-tically helpful in diminishing skin ruddiness and postinflammatory hyperpigmentation. Interestingly, it appears to be more useful for the hyperpigmen-tation related to skin aging (33).

*R. officinalis* leaves possess a variety of bioactive agents, including antioxidants and anti-inflamma-tories (34). The most potent antioxidant constituents are polyphenolics such as carnosic acid and carnosol (35). The results of this study showed that* R. officinale* has the protective effects in decreasing macromolecular damage in aged mice during aging. In addition, *R. officinale* extract has shown free radi-cal scavenging effect in the hippocampus (36). This is supported with a raising number of reports showing that natural extracts and phytochemicals have a constructive effect on brain aging through their action on ROS, specifically in the hippocampus (37).

*P. harmala* L. is known as Syrian rue, Wild rue and Harmal. The *P. harmala* has antibacterial, antifungal, antiviral, antioxidant, antidiabetic, antitumor, antileishmanial, insecticidal, cytotoxic, hepatopro-tective, and antinociceptive effects (38-39). In this study *P. harmala* showed the protective effects in improving antioxidant, anti-inflammatory and male sex hormones that were affected in aged mice during aging. In fact, flavonoids as a powerful antioxidant isolated from *P. harmala*, can remove the lipid peroxide radicals (40). Also, *P. harmala* treatment appeared to be a versatile strategy to conserve testicular uprightness and function during aging in male rats (41).

The leaves of *A. vera* (*A. barbadensis*) (Fam. Liliaceace) are the source of aloe vera gel. *A. vera* gel is greatly used in cosmetics and toiletries for its moisturizing and regenerating action. Also, the leaf of *A. vera *could assist cellular repairing, imbibition of foods, vitamins, minerals and vital nutrients (42). In this study* A. vera* showed a protective effect in improving antioxidant, anti-inflammatory and male sex hormones that were affected in aged mice. The anti-inflammatory property of *A. vera* has been documented in inflammation through suppression of free radicals and ROS (43). It has been shown that the life-long dietary supplementation of *A. vera* suppresses many age-related consequences in rats (44). Also, it has been suggested A. vera could suppress oxidative damage and age-related increases in hepatic cholesterol during life-long dietary (45).

*S. hortensis *L. is an annual culinary herb belonging to the family Labiatae. It is known as summer savory. Besides, this plant exhibited analgesic, antibacterial, antifungal, antioxidant and antihyperglycemic properties. In addition, the antigenotoxic effects of *S. hortensis *L. was shown on rat lymphocytes exposed to oxidative stress (46).The major constituents of the S. *hortensis* are carvacrol, gamma-terpinene, thymol and para-cymene (47). The investigation showed that carvacrol, thymol and flovonoids of Satureja spices are responsible to marked reduction of serum cholesterol in diabetic patients (48-50). It has been reported that age-related alterations of fatty acid composition in liver was accompanied by intake of savory essential oil through intensification of polyunsaturated fatty acids synthesis in mice liver and reduction of lipid peroxidation products (51). As a result, S. *hortensis* shows protective effects in improving antioxidant, anti-inflammatory and male sex hormones that were affected in aged mice during aging.

The genus *Teucrium *(Labiatae) comprises 12 species, which possess antioxidant, anti-spasmodic, anti-nociceptive and anti-inflammatory properties (52-54). According to the literature, *b*-caryophyllene and caryophyllene oxide were reported as the main sesquiterpenes in many *Teucrium *species. Investigation of the chemical constituents of the oil of *T. scordium *showed *b*-caryophyllene, caryo-phyllene oxide and also (*E*)-*b*-farnesene as the major components of* T. scordium *(55). In addition, *b*-caryophyllene is known as anti-inflammatory sesquiterpene (56); this effect may confirm the anti inflammatory activity of this plant (55). Nevertheless, no reports are valid about *T. scordium* in relation to its possible oxidative stress inhibitory potential in aged individuals. As much as we know this is the first study that shows antioxidant and anti-inflammatory effects of *T. scordium* extract in the aged rats. Our results indicated that *T. scordium* decreases inflammatory mediators and increases anti-oxidative power and steroid hormones in aged mice. Thus, *T. scordium* could be included in the diet as a nutritional supplement to increase the defenses of body against oxidative stress.

*H. perforatum*L is responsible for pharmacological properties, antiseptics (57), anti-inflammatory (58), antitumoral activities (59, 60). Today,* H. perforatum* is known as one of the few economic plants that include great ingredients of hypericins, hyperforins, and flavonoids. The results of this study showed that* H. perforatum *owns the protective effects in decreasing macromolecular damage in aged mice during aging. St. John’s wort is the dried tops or aerial parts of *H. perforatum* which gathered before or during flowering and is used in the therapy of anxiety related to aging (61).

Silymarin (SM), a flavonoids complex known as ‘milk thistle’ is extracted from the fruit of *Silybum marianum* (L.) Gaertn. (Carduusmarianus L., Astera-ceae). Interestingly, the present findings confirmed that *S. marianum* causes the best effects in improving antioxidant, anti-inflammatory and male sex hormones in aged mice. This effect is so important and should be considered as an advantage. This can be explained with current knowledge that among many medicinal plants, *S. marianum*, has been greatly used for centuries as a natural popular complementary medicine for the treatment of several diseases. The main indications for the use of silymarin are related to the hepatoprotection (62, 63). Also, its efficacy in inflammatory oxidative-mediated diseases like colitis has been confirmed (64, 65). It is noteworthy that several age-related brain and neurodegenerative diseases happen due to amplified oxidative stress. The search for compounds acting on the upgrading of cognitive performance and neuroprotection through antio-xidant motion is now a great interest (66). Excitingly, it has been shown that use of *S. marianum* for prevention and treatment of neurodegenerative diseases and processes associated with aging improves physiological responses against the ROS in the neural cells (67). 

## Conclusion

Taking collectively, the present results confirmed our hypothesis that the herbs with highest antioxidant power may reduce speed and rate of aging as evidenced by recovery of proinflammatory cytokines and sex hormones. Among tested herbs, S. marianum showed the best effect in improving all the D-galactose-induced aging effects. Since all of the selected and examined herbs are already found safe in human and there are good information from traditional medicine, therefore, they can be supplemented into the diet of elderly people to reduce speed of aging. Testing the mixture of these herbs together or with other anti-aging products is among the plans of future.
